# Endoskeletal mineralization in chimaera and a comparative guide to tessellated cartilage in chondrichthyan fishes (sharks, rays and chimaera)

**DOI:** 10.1098/rsif.2020.0474

**Published:** 2020-10-14

**Authors:** Ronald Seidel, Michael Blumer, Júlia Chaumel, Shahrouz Amini, Mason N. Dean

**Affiliations:** 1 B CUBE—Center for Molecular Bioengineering, Technical University Dresden, 01307 Dresden, Germany; 2 Max Planck Institute of Colloids and Interfaces, Department of Biomaterials, 14424 Potsdam, Germany; 3 Medical University Innsbruck, Division of Clinical and Functional Anatomy, 6020 Innsbruck, Austria

**Keywords:** cartilaginous fish, tessellated cartilage, tesserae, vertebrate endoskeleton, biomineralization

## Abstract

An accepted uniting character of modern cartilaginous fishes (sharks, rays, chimaera) is the presence of a mineralized, skeletal crust, tiled by numerous minute plates called tesserae. Tesserae have, however, never been demonstrated in modern chimaera and it is debated whether the skeleton mineralizes at all. We show for the first time that tessellated cartilage was not lost in chimaera, as has been previously postulated, and is in many ways similar to that of sharks and rays. Tesserae in *Chimaera monstrosa* are less regular in shape and size in comparison to the general scheme of polygonal tesserae in sharks and rays, yet share several features with them. For example, *Chimaera* tesserae, like those of elasmobranchs, possess both intertesseral joints (unmineralized regions, where fibrous tissue links adjacent tesserae) and recurring patterns of local mineral density variation (e.g. Liesegang lines, hypermineralized ‘spokes’), reflecting periodic accretion of mineral at tesseral edges as tesserae grow. *Chimaera monstrosa*'s tesserae, however, appear to lack the internal cell networks that characterize tesserae in elasmobranchs, indicating fundamental differences among chondrichthyan groups in how calcification is controlled. By compiling and comparing recent ultrastructure data on tesserae, we also provide a synthesized, up-to-date and comparative glossary on tessellated cartilage, as well as a perspective on the current state of research into the topic, offering benchmark context for future research into modern and extinct vertebrate skeletal tissues.

## 1. Introduction

**Figure 1.**
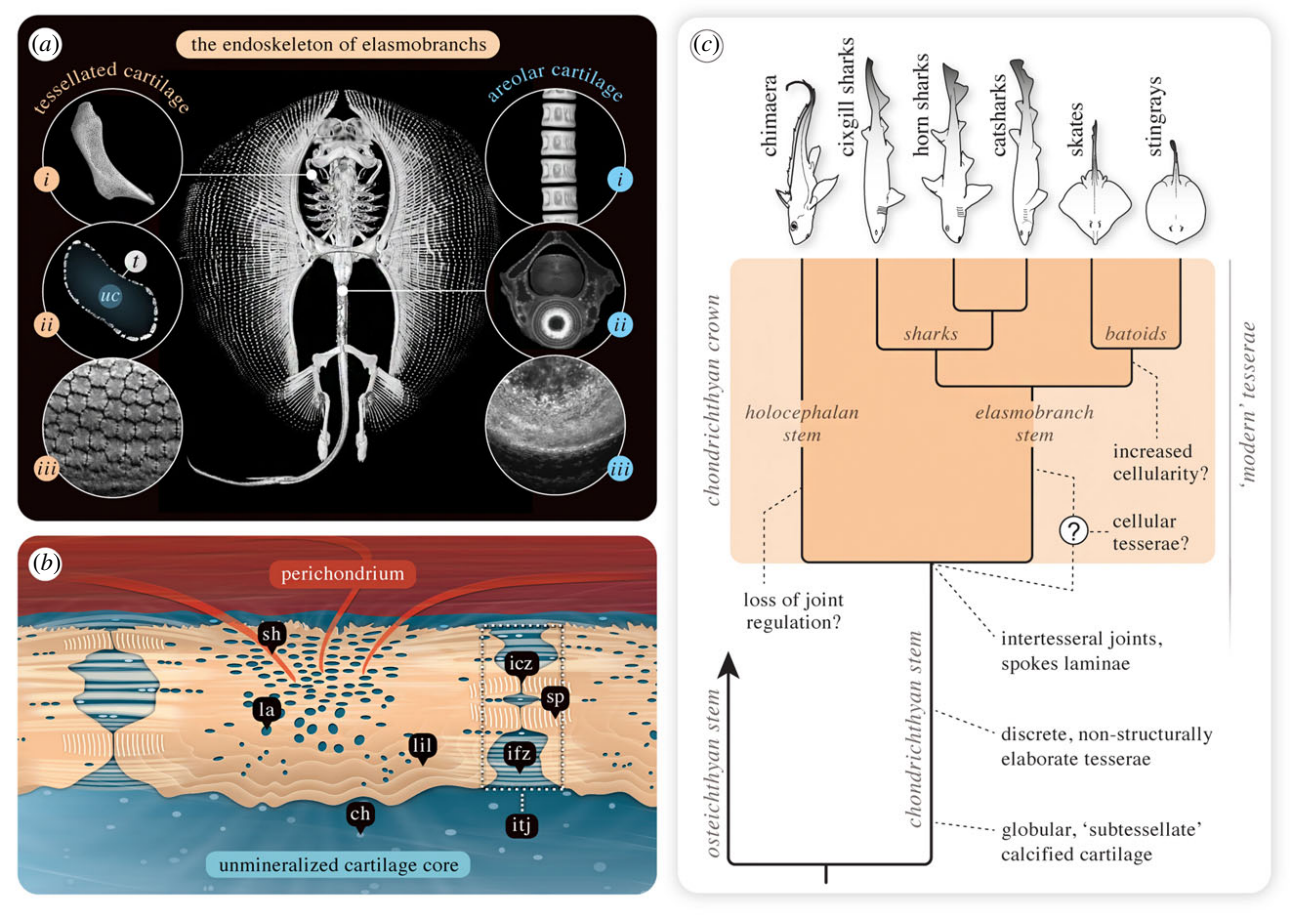
Mineralized skeletal tissue in sharks and rays (Elasmobranchii) and the evolution of tessellated cartilage in cartilaginous fishes. (*a*) Sharks' and rays’ cartilaginous skeletons show two primary mineralized tissues, tessellated and areolar cartilage. Tessellated cartilage: comprises most of the endoskeleton, where the unmineralized skeletal core is sheathed in a mineralized and tessellated layer, shown here in the round stingray *U. halleri* and (i) its hyomandibula, a skeletal element connecting the jaws and cranium. (ii) Cross-sections of elasmobranch skeletal elements show the outer tessellated layer (t) is quite thin, with the bulk of the skeletal cross-section being unmineralized, hyaline-like cartilage (uc). (iii) In modern sharks and rays, the individual tesserae are typically small (less than or equal to 500 µm across), polygonal and numerous—for example, this hyomandibula is covered by thousands of tesserae. Areolar cartilage: a less studied and less broadly distributed tissue, found exclusively in the spool-shaped centra of (i) the vertebral column. (ii) A cross-section of a vertebra, showing a tessellated neural arch topping the (iii) centrum, with mineralized tissue concentric around the centrum's core (notochordal remnant). (*b*) A schematic cross-section of tessellated cartilage showing tesserae are closely associated with various types of connective tissue, sandwiched between a fibrous perichondrium and the cartilaginous skeletal core. (*c*) Condensed chondrichthyan phylogeny, including stem and crown groups and reflecting the current state of knowledge of the evolution of specific structural features of tessellated cartilage. The more elaborate ‘modern’ tesserae (see (*b*)) described in crown chondrichthyans appear to have been derived from simpler mineralized tiles in stem chondrichthyans. The difficulty determining phylogenetic affinities for extinct taxa (particularly stem chondrichthyans), the patchy phylogenetic record of tesserae from fossil species and the lack of broad comparative data for modern species currently limit our understanding of the precise occurrence of ‘modern’ tesserae and their ecological significance in chondrichthyans. Modern species data derived from the current work and studies cited in the text; fossil data and phylogeny synthesized from [2]. All images in (a) from *Urobatis*, except areolar cartilage, (i), from a tiger shark. All images from μCT scan data, except areolar cartilage, (ii and iii) from confocal fluorescence microscopy. See text for explanation of features; ch, chondrocytes; icz, intertesseral contact zone; ifz, intertesseral fibre zone with aligned fibre bundles linking tesserae; itj, intertesseral joint = icz + ifz; la, cell lacunae; lil, Liesegang lines; sh, Sharpey's fibres; sp, spokes. (Image (*b*) adapted from [3] with permission from Elsevier.)

The cartilaginous fishes—sharks, rays (Elasmobranchii) and closely related chimaera (Holocephali)—exhibit a uniting anatomical character: unmineralized cartilage forms the majority of the skeleton of both juvenile and adult animals. This is an extraordinary model in the field of skeletal biology, since skeletal development in nearly all other vertebrate animals involves replacing the unmineralized embryonic cartilage skeleton with a mineralized bony one [1]. The skeletons of most cartilaginous fishes do mineralize, but in a quite different fashion: the majority of the skeleton (except some regions of the vertebrae) is stiffened only by a thin, outer layer of mineralized tissue, typically a few hundred micrometres thick (figure 1*a,b* and table 1). This outer crust is not continuous, but rather is broken into a mosaic of multitudinous minute polygonal tiles, called tesserae. This mineralized and tessellated covering of skeletal cartilage has been stated to be ‘*the* critical defining character’ for the entire group of modern cartilaginous fishes (Chondrichthyes) [84] but, as we show below, this is curiously still a subject of debate. The second major type of skeletal mineralized tissue in cartilaginous fishes is the *areolar* mineralized cartilage in the vertebral centra (figure 1*a*), but in contrast with tessellated cartilage, its structure and development have received little attention. *Lamellar mineralization*, an additional, poorly known skeletal type of mineralization with some bone-like qualities, sheathing the neural arches, has to date only been observed in some carcharhiniform sharks [9,28].

**Table 1.** Glossary of published articles about tesserae and tessellated cartilage from extant sharks and rays (Elasmobranchii). The table is subdivided by topic; studies either focus on the listed topic or include substantial statements about them.

keyword	publication
general reviews of tessellated cartilage structure and function	Applegate [4]; Dean & Summers [5]; Maisey [6]; Dean *et al*. [7]; Dean [8]; Debiais-Thibaud [9]; Huber *et al*. [10]; Seidel *et al*. [11,12,13]; Maisey *et al*. [2]
juvenile tesserae and tesserae development	Benzer [14]; Ørvig [15]; Schmidt [16]; Bordat [17]; Clement [18]; Kemp & Westrin [19]; Eames *et al*. [20]; Dean *et al*. [21]; Maisey, [6]; Enault *et al*. [22]; Seidel *et al*. [23]
factors regulating tesserae mineralization	*alkaline phosphatase:* Lorch [24]; Urist [25]; Eames *et al*. [20]; Omelon *et al*. [26]; Atake *et al*. [27] *collagen Type X:* Seidel & Blumer *et al.*, [3]; Debiais-Thibaud *et al.* [28] *proteoglycans:* Takagi *et al*. [29]; Gelsleichter *et al*. [30]; Egerbacher *et al*. [31] *peptides/proteins:* Glowacki *et al*. [32]; Trivett *et al.* [33]; Ortiz-Delgado *et al*. [34]; Egerbacher *et al*. [31]
cells and lacuno-canalicular networks in tesserae	Müller [35]; Leydig [36,37]; Tretjakoff [38]; Lorch [24]; Ørvig [15]; Applegate [4]; Peignoux-Deville *et al*. [39]; Bordat [17]; Clement [18]; Summers [40]; Ortiz-Delgado *et al*. [34]; Egerbacher *et al*. [31]; Dean *et al*. [21,41,42]; Johanson *et al*. [43]; Maisey [6]; Omelon *et al*. [26]; Seidel *et al*. [23,44]; Knötel *et al*. [45]; Seidel & Blumer *et al*. [3]; Huang *et al.*, [46]; Atake *et al*. [27]; Chaumel *et al*. [47]; Marconi *et al*. [48]
collagen types in elasmobranch skeletal tissues	Peignoux-Deville *et al*. [39]; Rama & Chandrakasan [49]; Takagi *et al*. [29]; Sivakumar & Chandrakasan [50]; Mizuta *et al*. [51]; Egerbacher *et al*. [31]; Eames *et al*. [20]; Enault *et al*. [22]; Seidel & Blumer *et al*. [3]; Debiais-Thibaud [28]; Atake *et al*. [27]
elemental composition of tesserae	Marchand [52]; Urist [25]; Huang *et al*. [46]; Seidel *et al*. [9,44]
intertesseral joints anatomy (incl. fibres and cells)	Roth [53]; Tretjakoff [38]; Bargmann [54]; Kemp & Westrin [19]; Bordat [17]; Clement [18]; Summers [40]; Maisey [6]; Knötel *et al*. [45]; Seidel *et al*. [3,12,23,44]
fusion of tesserae in elasmobranchs	Ridewood [55]; Applegate [4]; Kemp & Westrin [19]; Maisey [6]
Liesegang lines—wave-like mineral density variations	Tretjakoff [38]; Weidenreich [56]; Bargmann [54]; Ørvig [15]; Applegate [4]; Moss [57]; Kemp & Westrin [19]; Peignoux-Deville *et al*. [39]; Takagi *et al*. [29]; Bordat [17]; Johanson *et al*. [43]; Seidel *et al*. [12,23,44]; Dean *et al*. [58]; Seidel *et al*. [3,13]
tesserae micro-mechanical properties	Wroe *et al*. [59]; Seidel *et al*. [13]; Jayasankar *et al*. [60]
tessellated cartilage mechanical properties	Fahle & Thomason [61]; Liu *et al*. [62,63]; Ferrara *et al*. [64]; Summers [40,65]; Macesic & Summers [66]; Mulvany & Motta [67]; Balaban *et al*. [68]; Wilga *et al*. [69]; Huang *et al*. [46]; Jayasankar *et al*. [60,70]; Rutledge *et al*. [71]; Seidel *et al*. [12]
Sharpey's fibres—perichondral fibres in tesserae	Bargmann [54]; Ørvig [15]; Kemp & Westrin [19]; Seidel & Blumer *et al*. [3]
spokes—structural, tesserae reinforcements	*first description:* Seidel *et al*. [23] *figured in:* Tretjakoff [38]; Kemp & Westrin [19]; Lee *et al*. [72]; Bordat [17]; Ortiz-Delgado *et al*. [34]; Johanson *et al*. [43]; Seidel *et al*. [3,12,13,23,44]; Huang *et al*. [46]; Atake *et al*. [27]; Chaumel *et al*. [47]; Jayasankar *et al*. [60]; Maisey *et al*. [2]
supra-tesseral cartilage layer	Lorch [24]; Roth [53]; Bordat [17]; Clement [18]; Egerbacher *et al*. [31]; Maisey [6]; Enault *et al*. [22]; Seidel & Blumer *et al*. [3]
tesserae patterning network	Knötel *et al*. [45]; Jayasankar *et al*. [60,70]; Baum *et al*. [73]; Seidel *et al*. [13]
multiple, layers of tessellated cartilage	Dingerkus [74]; Maisey [6]; Dean *et al*. [58]; Seidel *et al*. [12,44]; Maisey *et al*. [2]
trabeculae – jaw cartilage reinforcement	Bargmann [54]; Summers *et al*. [65,75]; Summers [40]; Dean *et al*. [5]; Dean [8]; Seidel *et al*. [11,12,44]; Rutledge *et al*. [71]
tessellated cartilage repair in elasmobranchs	Glowacki *et al*. [76]; Clement [77]; Ashhurst [78]; Dean *et al*. [58]; Seidel *et al*. [44]; Marconi *et al*. [48]
inter- and intraspecies variation of tesserae shape	Roth [53]; Maisey [6]; Seidel *et al*. [23]; Atake *et al*. [27]; Jayasankar *et al*. [60,70]
evolution of tessellated cartilage	Ørvig [15]; Halstead [79]; Moss [57,80]; Maisey [6]; Donoghue & Aldridge [81]; Donoghue & Sansom [82]; Donoghue *et al*. [83]; Enault *et al*. [22]; Debiais-Thibaud [9]; Maisey *et al*. [2]

Elasmobranch tessellated cartilage has been a topic of interest for comparative anatomists and palaeontologists since the early 1800s, however there has been a sharp increase in attention recently. In the last two decades alone, the number of publications focused on tessellated cartilage has tripled, drawing new interest from the fields of biomaterials, molecular biology and evolutionary genetics (table 1). The diverse multidisciplinary appeal of shark and ray cartilage springs from a variety of factors. From engineering and materials science perspectives, tesserae are believed to be a key feature in enhancing the mechanical performance of the otherwise flexible cartilage skeleton [13,60,62,63,70]. Evolutionary geneticists and skeletal biologists are motivated by tessellated cartilage's distinct tissue characteristics relative to mammalian cartilage and its role as a functional alternative to bone in vertebrates. At the same time, palaeontologists and evolutionary biologists have benefitted from the increase in research on modern forms (figure 1*c*), as they have allowed comparisons with extinct elasmobranchs, where teeth, vertebrae and tesserae are some of the few things that fossilize. These comparisons have suggested that the tesserae as we know them from modern sharks and rays first appeared at some point in stem chondrichthyans (e.g. the extinct shark *Mcmurdodus*), derived from the ‘simpler’ (i.e. less structurally elaborate) tesserae seen in earlier stem chondrichthyan species (e.g. the extinct, shark-like *Doliodus*) [2,85–87].

In contrast with elasmobranchs, the fossil record for chimaera, the sister group to modern sharks and rays, is fragmentary. This has been partly explained by evolutionary shifts towards a deeper water lifestyle, into regions with lower potential for fossilization [84], but could also be a function of the skeletal tissue being relatively poorly mineralized. As a result of the patchy fossil record, the evolution of holocephalan skeletal tissues, but also of the degree and morphology of mineralization is not well understood. Some palaeontological work shows that early holocephalans (ancestors of modern chimaera) did exhibit skeletal polygonal tessellations [88,89]. Yet, the limited literature on skeletons of modern species is contradictory, raising the question whether tesserae (and some of their specific features) are present in modern chimaera at all [2,9,37,53,89–91]. Understanding the nature of skeletal mineralization in chimaera will provide a missing perspective on the biology of this enigmatic group, and is necessary to clarify character states, phylogenetic affinities and selective factors associated with the ancient phylogenetic split within Chondrichthyes, which yielded the two major, modern groups of cartilaginous fishes (figure 1*c*).

We provide here the first ultrastructural description of a modern chimaeroid skeleton, investigating skeletal mineralization in the holocephalan *C. monstrosa*. We apply modern, high-resolution and three-dimensional imaging techniques to the study of chimaera skeletal tissue, the same characterization tools that fuelled part of the massive upsurge in elasmobranch tessellated cartilage research in the past decade (table 1). In the following sections, we draw on the available data for elasmobranch tessellated cartilage to detail characteristic structural and material features that, to the best of current knowledge, define what tesserae are—from their morphology to their materials—and how they vary in modern elasmobranchs. Since the only detailed information on modern tesserae comes from elasmobranchs, for each feature, we summarize elasmobranch data first to provide a standard for interpreting our observations on the skeletal tissue of *C. monstrosa*. Additionally, we compile an in-depth glossary for tessellated cartilage, synthesizing data across published work for the last greater than 150 years. In these ways, the current work is an overdue update to the most cited paper on tessellated cartilage —Dean and Summers' 2006 review— detailing features that were discovered in the past decade, some of which have proved to be unifying features of elasmobranch tesserae, and framing open questions for future work. In this article, we focus entirely on tessellated cartilage: besides work on the structure, composition and mechanics of vertebrae [92–94], our knowledge of vertebral areolar mineralization has hardly progressed since Dean and Summers' review [5]. The lamellar mineralization seen in carcharhiniform shark vertebrae is even less known; see [9] for a synthesis of the scant current data.

## 2. What is a tessera? Defining features of tessellated cartilage

Over the past two decades, skeletal calcification in elasmobranchs has been examined at several hierarchical levels and scales, in a variety of species, but particularly in three that have become research models: a catshark (*Scyliorhinus canicula*), stingray (*Urobatis halleri*) and skate (*Leucoraja erinacea*) (table 1). Previously, comparison and interpretation of findings across studies were challenged by the diversity of tesserae morphologies (e.g. interspecifically) [23]; the examination of different skeletal elements and species; and the varying sectioning planes and methods used to study tesserae and adjacent tissues (e.g. unmineralized cartilage, perichondrium). Even a small skeletal element can be covered by thousands of tesserae, which also show regional differences in shape and size [45,73], making it challenging to draw general conclusions about tissue structure. However, despite immense variation observed in tesseral morphology and tiling pattern (e.g. [6,23]), a number of characters appear to be common to the tesserae of different modern shark and ray species [23,44].

### 2.1. General morphology

#### 2.1.1. Elasmobranchs

**Figure 2.**
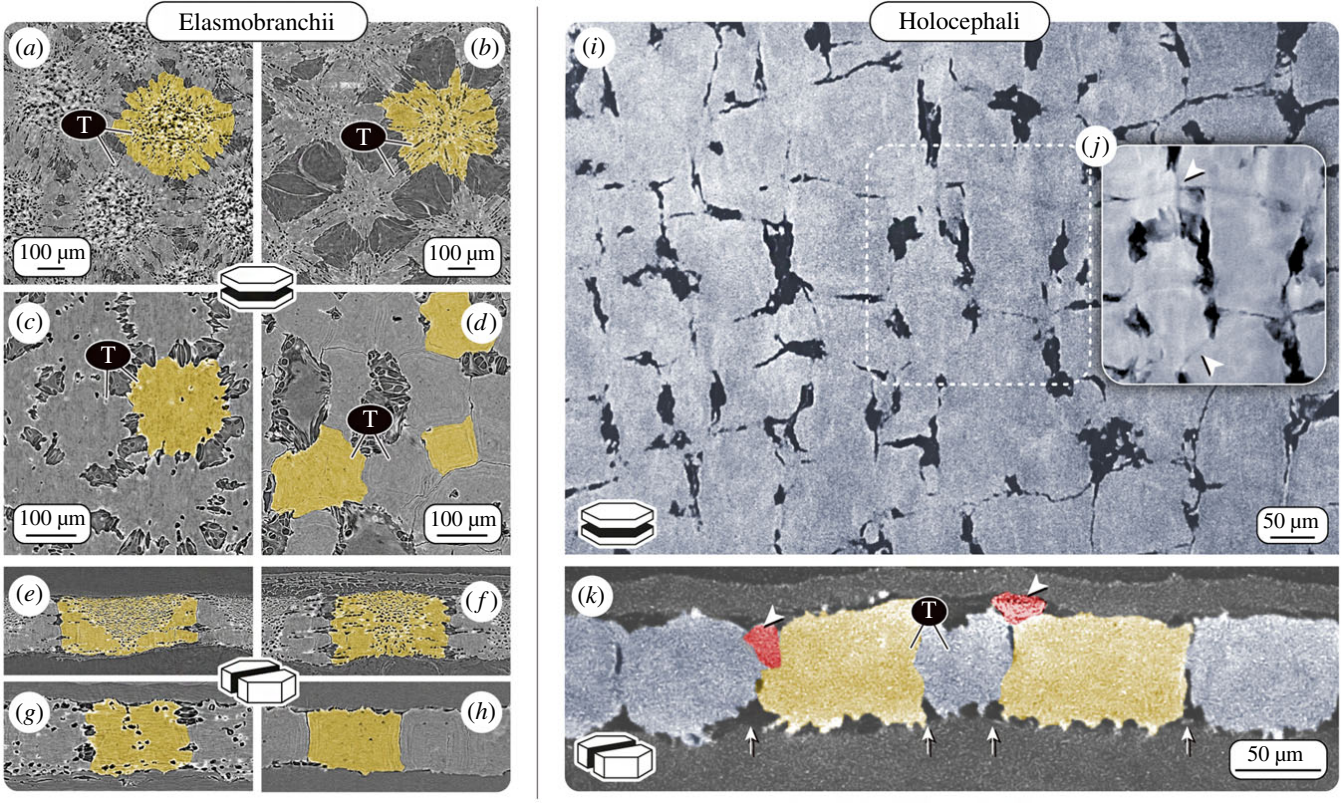
Variation in tessellation patterns in elasmobranchs and holocephalans. Computed tomography virtual sections of tesserae (T; with individual tesserae coloured yellow) from four different elasmobranch species: (*a,e*) a skate, *Raja stellulata*; (*b,f*) a stingray, *Pteroplatytrygon violacea* and two sharks (*c,g*) *Heterodontus francisci*; (*d,h*) *Notorynchus cepedianus*; and a holocephalan species (*i–k*) *C. monstrosa.* Images *a–d* and *i,j* show virtual planar sections, *e–h,k* are virtual vertical sections of tesserae. (*i*) The tiling pattern of skeletal mineralization in the chondrocranium of *C. monstrosa* appears most similar to that of the broadnose sevengill shark *Notorynchus cepedianus* (*d,h*), with tesserae exhibiting irregular geometric shapes and almost no cellular lacunae (visible as black dots in most of the elasmobranch tesserae). The presence of cell lacunae is considered the standard condition for (*a–h*) elasmobranch tesserae. (*j*) Averaged planar section created from six consecutive sections, revealing tesserae borders (intertesseral joints), which were not clearly visible in (*i*) a single section plane. (*k*) In vertical sections, some adjacent tesserae in *C. monstrosa* overlap (red tesserae, marked with arrowheads), whereas others are in contact at their lateral edges (intertesseral joints, marked with arrows). (Images *a–h* adapted from [23] with permission from Wiley.)

In sharks and rays, tesserae in adult animals can vary nearly an order of magnitude in width from, for example, approximately 100 µm in a catshark [23] to nearly 1 mm in large sawfish and guitarfish [44,58], although the functional and biological significance of different tesserae sizes across species is not yet understood. The great variation of tesserae sizes across ontogeny is owed at least partly to the fact that tesserae grow by accretion as the animals age and so smaller tesserae are likely more recently formed [21,23]. Shape variation of tesserae has also not been broadly characterized or quantified; however, on a 2 cm piece of skeleton from a stingray (the hyomandibula of *U. halleri*), nearly 50% of the 2768 tesserae were six-sided, ranging from four- to eight-sided geometries [73]. Although tesserae are often depicted as polygonal, they can also be more stellate in their surface aspect [27,60] and can range from thin plates to cubes to tall columnar blocks in their vertical cross-sections (figure 2*a–h*) [2,12]. In general, in adult animals, tesserae are typically wider than deep with dimensions of several hundred micrometres (e.g. in *U. halleri*, approx. 500 µm and approx. 300 µm in width and height, respectively; [21,23,73]). The tesseral layer is often thicker or even multi-layered in skeletal regions that experience high loads (e.g. beneath the tooth plates of rays that eat hard prey) [5,6,11,44,58,74]. In the round stingray *U. halleri*, for example, the shape aspect ratio of tesserae can also be inverted at the highly curved jaw margins, resulting in a thick mineralized crust of columnar tesserae far taller than wide.

#### 2.1.2. Chimaera

Our high-resolution micro computed tomography (μCT) imaging of the braincase of *C. monstrosa* shows that tesserae are indeed present, forming a thin layer of mineralized tissue just beneath the surface of the skeleton, overlain by a thin layer of unmineralized cartilage. Virtual planar and vertical sections through the calcified layer show tessellated regions interspersed with regions of unmineralized cartilage, the tesserae approximately 50–100 µm wide and approximately 50–75 µm thick, considerably smaller than most elasmobranch tesserae we have observed (figure 2*i*–*k*). *Chimaera monstrosa* tesserae are less regular in shape and size in comparison to the general scheme of polygonal tesserae that has proved largely typical in elasmobranchs. Tesserae in *C. monstrosa*, however, appear to be mostly block-like, almost cubic, most similar to tesserae from two members of probably the oldest lineages of modern sharks: the horn shark (*Heterodontus francisci*, figure 2*c*,*g*) and the broadnose sevengill shark (*Notorynchus cepedianus*, figure 2*d*,*h*) [23]. *Chimaera monstrosa* tesserae are packed closely together, often with no intertesseral gaps distinguishable in our tomography scans (spatial resolution: approx. 1.4 µm; voxel size: 452 nm). This tight packing is similar to elasmobranch tesserae [23,45], which abut yet typically do not overlap (figure 2*a*–*h*). By contrast, in *C. monstrosa*, some adjacent tesserae appear partly overlapped or fused, making it difficult to unambiguously define tesseral shapes and margins (figure 2*k*). We discuss the implication of this difference to elasmobranchs in more detail below.

### 2.2. Liesegang lines

#### 2.2.1. Elasmobranchs

**Figure 3.**
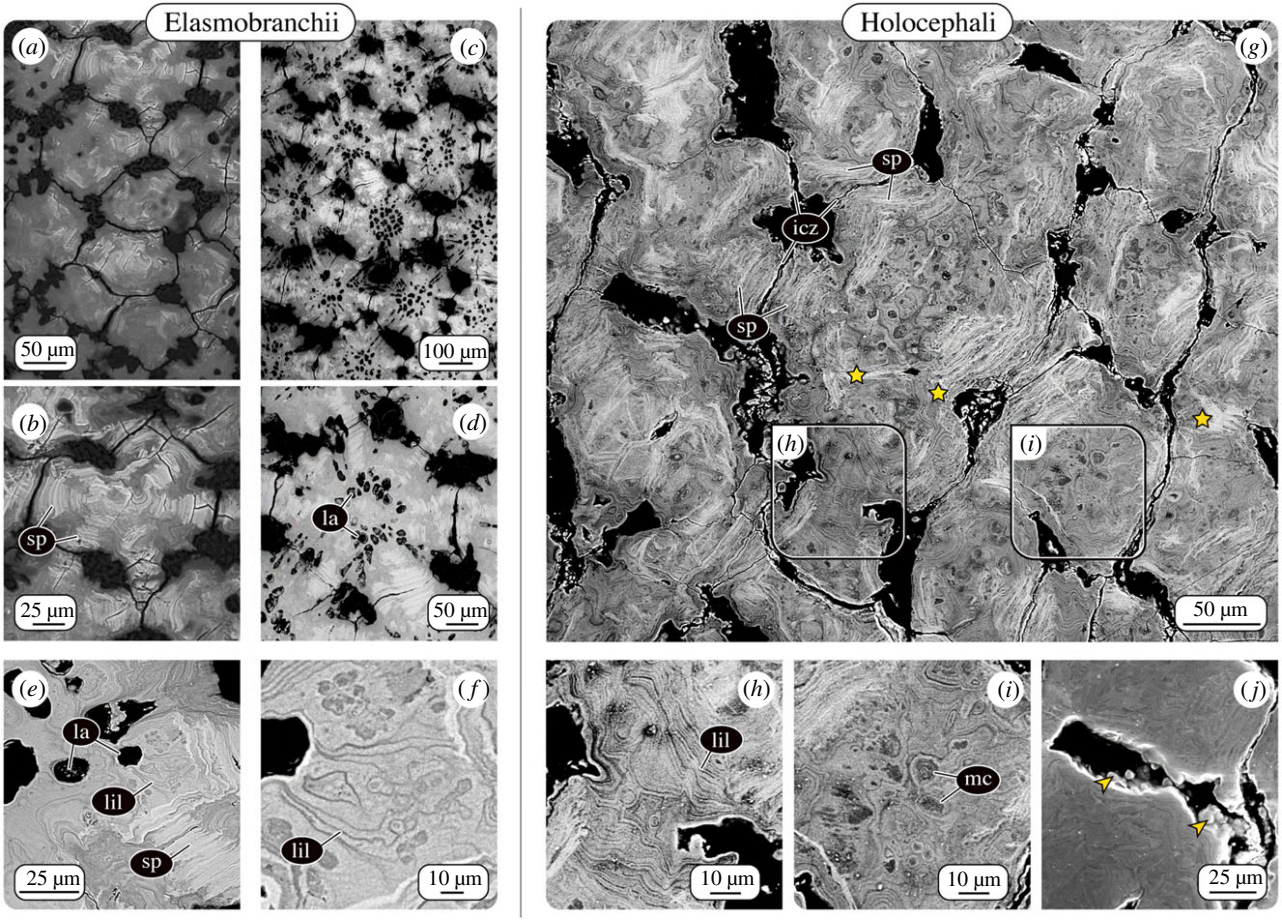
Patterns of mineral density variation in tesserae. BSE allows visualization of differences in either tissue elemental density (e.g. mineral density) or elemental composition as variation in greyscale values (low and high concentrations shown as dark and bright grey values, respectively). Although tesserae from (*a,b)* chain catshark *Scyliorhinus retifer*, (*c–f) U. halleri* and (*g*–*j*) *C. monstrosa* differ in their morphology and tiling pattern, they show similar intratesseral features of varying mineral density. All three species exhibit acellular spokes (sp), high mineral density features in regions where adjacent tesserae abut one another (intertesseral contact zones, icz). Unlike in the elasmobranch tesserae, in some regions, in *Chimaera* (*g*), adjacent tesserae appear fused (stars). Liesegang lines (lil), successive growth lines reflecting accretional growth in elasmobranchs; (*e*) Liesegang lines (lil) in tesserae from *U. halleri*, are also visible in (*h*) tesserae from *C. monstrosa*. (*g–i*) Unlike the majority of elasmobranch tesserae, tesserae in *C. monstrosa* lack cell lacunae (la). Dark, ovoid regions, however, suggest the presence of mineralized cells (mc) or in-filled cell lacunae. (*j*) Mineralized globules associated with tesseral borders (arrowheads) suggest similar mechanisms of accretional growth between elasmobranch and chimaera tesserae [23].

Local variation in mineral density—the amount of mineral packed into a tissue—is a hallmark of bio-mineralized vertebrate tissues and a visual record of their growth. In bone, for example, the mineral density variation can be used to identify tissue that has been newly deposited or remodelled [95–97]. Similarly, elasmobranch tesserae, are also not merely homogeneous blocks of mineral: when sectioned they show swirling, concentric patterns of varying mineral density (Liesegang lines; figure 3), radiating out from the centres of tesserae and tracking the shape of adjacent features like tesseral edges or cell lacunae (see below). Although the concentric patterning of Liesegang lines was noted by authors as early as the late 1800s, only recently, backscattered SEM imaging (BSE) and energy dispersive X-ray spectroscopy (EDX) clarified that the patterning results purely from variations in mineral density, rather than variation in chemical composition. Further, Liesegang lines provide a record of growth in tessellated cartilage, representing successive events of newly mineralized matrix accreted onto tesseral edges [3,23]. Intersections of consecutive Liesegang lines or abrupt cessation of the pattern have not been observed in elasmobranch tesserae [23], arguing that the tissue is never locally remodelled. This is in contrast with osteonal bone, where newer osteons intersect and interrupt older ones, providing a roadmap of remodelling activity [98].

#### 2.2.2. Chimaera

Liesegang lines are also prominent in the tesserae of *C. monstrosa*, as characteristic bands of different mineral density without any visible intersections (figure 3). This points to a consecutive and deposition-only mineralization process in *C. monstrosa* tesserae, as in elasmobranch tesserae. In fact, in planar sections through the tessellated layer of *C. monstrosa*, the concentric Liesegang lines form relatively large-scale patterns, in the form of long continuous lines at the edges of tesserae (figure 3*h*). This suggests that adjacent tesserae originated quite far apart, before gradual mineral accretion eventually brought them into contact. Where tesserae have grown together, we see two very different morphologies: either individual tesserae in direct contact, with the flanking tissue reinforced by distinct, higher mineral density features (spokes, see below), or tesserae that have apparently fused, the Liesegang lines originating from the different tesserae colliding in an incongruous pattern (figure 3*h*). The functional separation of tesserae is believed to be central to the mechanical behaviour of tessellated cartilage [12,60,99]. We have never observed such complete fusions of adjacent tesserae in elasmobranchs; however, other authors have reported these in some species [4,6]. Adjacent elasmobranch tesserae occasionally appear linked by mineralized perichondral collagen on their outer edges, obliterating the tessellation pattern on the skeletal surface, in some cases in regions of apparent previous damage (e.g. fig. 4a in [44]; fig. 5 in [58]). This suggests that there are mechanisms that control the maintenance of gaps between tesserae in elasmobranchs and that these can break down under certain (yet unknown) conditions. The partial and apparently recurrent fusion of tesseral edges in adult *C. monstrosa* suggests the mechanisms that maintain the separation of tesserae may differ or be absent in chimaera. This may also explain why some authors described modern chimaera skeletal mineralization as ‘continuous’ [90] or ‘granular’ [89], but not tessellated.

### 2.3. Spokes

#### 2.3.1. Elasmobranchs

Early in development, incipient tesserae form at some distance from one another, separated by unmineralized matrix. As a result of their accretive growth, they become larger and closer together over time [13,21,23]. It was only recently discovered that once tesserae grow into contact, they develop dramatic hypermineralized features called spokes (figures 1*b* and 3). These form exclusively in areas where adjacent tesserae abut; in adult animals, spokes radiate from the margins of large tesserae to their centres like spokes on a wheel. Spokes are characterized by thin (approx. 1–3 µm) laminae of alternating high and low mineral density, oriented parallel to the tessera edge [13,23] (figure 3*a*–*e*). To date, spokes have been present in all adult elasmobranch tesserae we have examined with BSE imaging regardless of species or tesseral shape variation. In BSE, spokes are probably the most conspicuous feature of (sectioned) adult tesserae, their very high mineral density similar to dental rather than skeletal tissues, making them far brighter than the rest of the tesseral body. The specific arrangement of spokes suggests they reinforce regions of collision between tesserae and transmit contact stresses away from cell-rich areas, hypotheses supported by recent modelling studies [13,23,60].

#### 2.3.2. Chimaera

**Figure 4.**
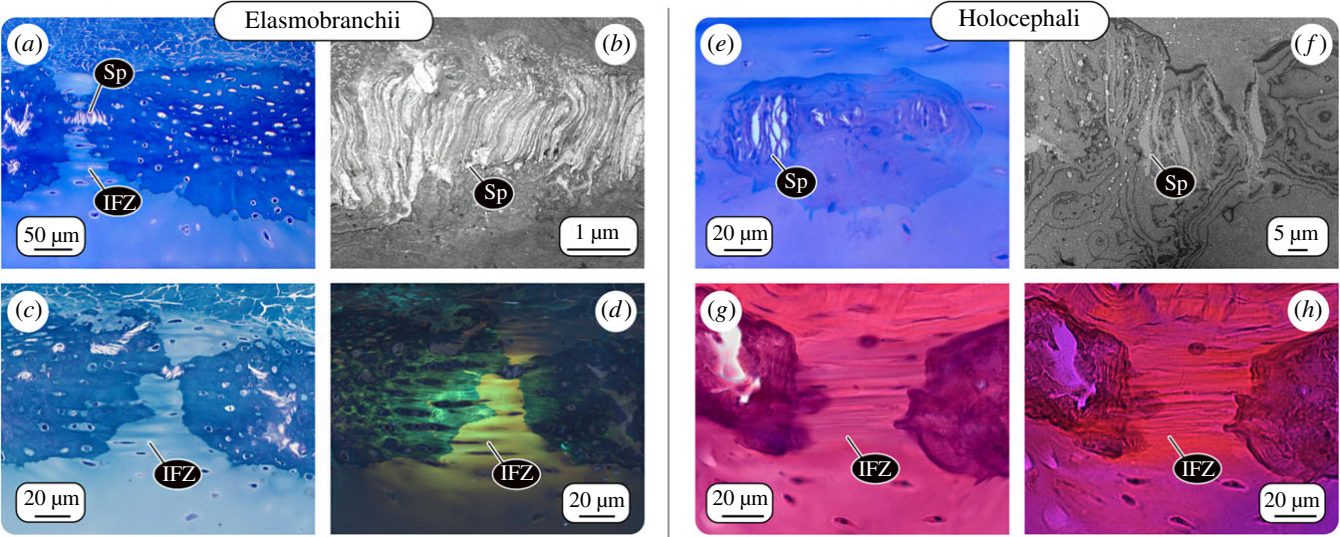
Intertesseral joints and spokes. LM and TEM of adjacent tesserae from an elasmobranch and chimaera. (*a,b,e–f*) In demineralized tesserae from both elasmobranch (*U. halleri*) and chimaera (*C. monstrosa*), spokes (Sp) are visible as laminated structures, their laminae parallel to the lateral edge of the tesserae where they abut one another. (*c,d,g,h*) Intertesseral fibrous zones (IFZ), gaps between tesserae filled with densely aligned fibre bundles linking adjacent tesserae*.* (*d*) Between the fibre bundles, cells are aligned in strings in elasmobranchs, whereas (*h*) in chimaera, very few cells were visible in the IFZ. Images: (*a, c* and *e*) LM images of toluidine blue and (*g*) H&E stained sections; (*d,h*) polarized LM images of (*c,g*), respectively; (*b,f*) TEM images.

In *C. monstrosa*, the tesserae also exhibit laminated, highly mineralized features that resemble elasmobranch spokes (figure 3). However, unlike the radially organized, and joint-associated spokes of elasmobranchs, chimaera spokes are more disorganized and seem to not follow distinct patterning. Spokes were not restricted to peripheral, abutting mineralization fronts, but were rather occasionally visible in more central regions or near tesseral edges associated with unmineralized cartilage (figure 4*e*,*f*). We believe this observation reflects the three-dimensional organization of chimaera tesserae: spokes observed in these unexpected regions could result from points of contact with overlapping/underlying tesserae out of the current sectioning plane (e.g. as with the red tiles in figure 2*k*). If the arrangement of spokes reflects the loading environment in tesserae (as believed for elasmobranch tesserae), the haphazard arrangement of spokes in chimaera tesserae suggests they may experience quite diverse loading orientations. *Chimaera montrosa* tesserae are comparatively thin and small, and overlapping them may serve to increase the stiffness of the calcified layer and the skeleton as a whole. Although we suspect, based on ultrastructural similarities, that *C. monstrosa ‘*spokes’ are homologous to those of elasmobranchs, their appearance suggests that, once more species are examined, another descriptor implying more disorder might be more apt for chimaera ‘spokes’.

### 2.4. Intratesseral cells

#### 2.4.1. Elasmobranchs

There is growing evidence that the chondrocytes (cartilage cells) of elasmobranch cartilage are peculiar; elasmobranch chondrocytes neither undergo the significant changes in cell volume (hypertrophy; e.g. [100]) nor the cell death or transdifferentiation seen in mammalian chondrocytes during the cartilage calcification involved in long bone formation. In elasmobranchs, chondrocytes are incorporated alive into tesserae as they grow, engulfed into gaps (lacunae) in the mineralized tissue [21,23,42,47] (figure 3*c*–*e*). As a result, elasmobranch tesserae can be surprisingly cellular: adult *U. halleri* tesserae contain approximately 100 000 chondrocytes per cubic millimetre, similar to the cell density of unmineralized cartilage in small mammals [47]. Chondrocytes within tesserae demonstrate their capacity for keeping matrix mineralization at a distance, maintaining a surrounding envelope of unmineralized cartilage [3,19,21,23,80]. In larger (i.e. older) tesserae from adult stingray *U. halleri*, some cell lacunae can be filled with a material of extremely high mineral density, suggesting the resident cell's death released an inhibition on mineralization [23]. From electron microscopy and high-resolution μCT data, it was discovered that lacunae within tesserae are connected via small passages (canaliculi), forming a continuous lacuno-canalicular network (LCN) filled with cells and unmineralized matrix. The similarity to the LCN in bone—albeit with fewer, shorter, and thicker passages—suggests that the cell population within tesserae could form an intercommunicating and even mechanosensory nexus [42,47]. Lacunar shape and the arrangement of the LCN vary according to the location within tesserae [47], perhaps reflecting both growth processes (e.g. how cells were incorporated) and local mechanical demands [3,19,23].

#### 2.4.2. Chimaera

In *C. montrosa*, the mineralized matrix is entirely devoid of lacunar spaces or other cavities that could house cells (figure 2*i*,*k*). In BSE images, however, darker (i.e. less mineralized), circular blotches are visible throughout the mineralized tissue. Based on their size (approx. 10 µm) and distribution (figure 3*g*,*i*), we believe these represent former chondrocyte lacunae, in-filled with low mineral density tissue. This argues that chimaera, unlike elasmobranchs, share with other vertebrates an association between the death of chondrocytes and cartilage calcification. The question arises whether the calcification begins in concert with chondrocyte death (e.g. they act as focal points for mineralization), or, as in elasmobranchs, begins between chondrocytes in the extracellular matrix; however, in chimaera with cells dying once, they are surrounded by mineralized matrix. Both options argue that *C. montrosa* chondrocytes may be incapable of inhibiting mineralization. The absence of patent cell spaces and living cells in *C. montrosa* tesserae could point to a fundamental biological difference with the mechanisms of skeletal mineralization observed in most elasmobranchs [23]. The size similarity between *C. monstrosa* chondrocytes and their in-filled tesserae lacunae indicates that cell hypertrophy does not precede mineralization, as in some bony vertebrate cartilages. The range of chondrocyte physiologies our data suggest for cartilaginous fishes deepens recent assertions that cartilaginous fishes could be unappreciated models for understanding the diversity of cartilage cell function and behaviour [47,48].

### 2.5. Intertesseral joints

#### 2.5.1. Elasmobranchs

Due to the complex edge topology of elasmobranch tesserae, regions where neighbouring tesserae are in direct contact are surrounded by small zones where tesserae are not touching; these gaps are filled with unmineralized extracellular matrix, including cells and densely packed fibre bundles (figures 3 and 4). The fibre bundles span the gap between opposing tesserae, linking them with a flexible joint (figures 1–3). This fibrous connection is thought to be vital to skeletal mechanics, providing a measure of flexibility to the skeleton by allowing tesserae to pull apart from each other when the skeleton is loaded in tension [11,60]. The cells in the joint space are often flattened and slender and aligned end-to-end in series between the fibres. Since those cell strings often appear to begin in one tessera and end in another (figure 4*a*–*d*), they may form a communicating link between the intratesseral networks of adjacent tesserae, but this has not been explored. The fibrous matrix between tesserae (at least in *Urobatis*) is composed of at least three different types of collagen (Type I, II and X), as well as an additional fibre type, which appears glossy in TEM images, but has yet to be identified [3].

#### 2.5.2. Chimaera

Our histological staining and TEM imaging of mineralized, tessellated cartilage from *C. monstrosa* show intertesseral joints similar to those in elasmobranchs: regions of unmineralized extracellular matrix between tesserae with intertesseral fibres linking adjacent tesseral edges (figure 4*g*,*h*). However, obvious intertesseral joint gaps occur less regularly between tesserae in *C. monstrosa*, and histological images of *C. monstrosa*'s fibrous joint zones reveal that comparatively few (if any) cells occupy the space between tesserae. In elasmobranchs, these cells are proposed to coordinate growth of the joint region, namely elongation of joint fibres and inhibition of tesserae fusion [23]. The lack of intratesseral joint cells may, therefore, explain the overlaps and fusions that we observed among chimaera tesserae (see above) and indicate an absence of cellular-controlled maintenance of the joints between tesserae.

### 2.6. Elemental composition and material properties

#### 2.6.1. Elasmobranchs

From a material standpoint, the collagens and mineral that build elasmobranch tesserae do not seem to be unusual relative to other vertebrate skeletal tissues, it is how they are combined and arranged that gives tesserae interesting properties. Recent material characterization studies (e.g. via Raman spectroscopy and EDX) confirmed that elasmobranch tesserae comprise carbonated apatite, the same mineral in bone and calcified cartilage in other vertebrates [44,101,102]. The range of mineral densities that are observed in tesserae overlaps with the range of values reported for bone and calcified cartilage, yet exceeds their maximum mineral densities [13,103]. This has implications for mechanical performance, as micro-mechanical properties are positively correlated with local mineral density in mineralized tissues. Indeed, the highest mineral density regions in tesserae (e.g. spokes) exhibit the highest indentation values for stiffness and hardness—exceeding those reported for bone and calcified cartilage—and are localized to mechanically important tesseral regions (intertesseral contact zones) [13]. The exceptionally high local mechanical properties and densely packed mineral in some regions of tesserae indicate different tissue organization and growth processes relative to bone and calcified cartilage in other vertebrates [3,44], but the processes involved in shaping these tissue properties during development remain unexplored.

#### 2.6.2. Chimaera

**Figure 5.**
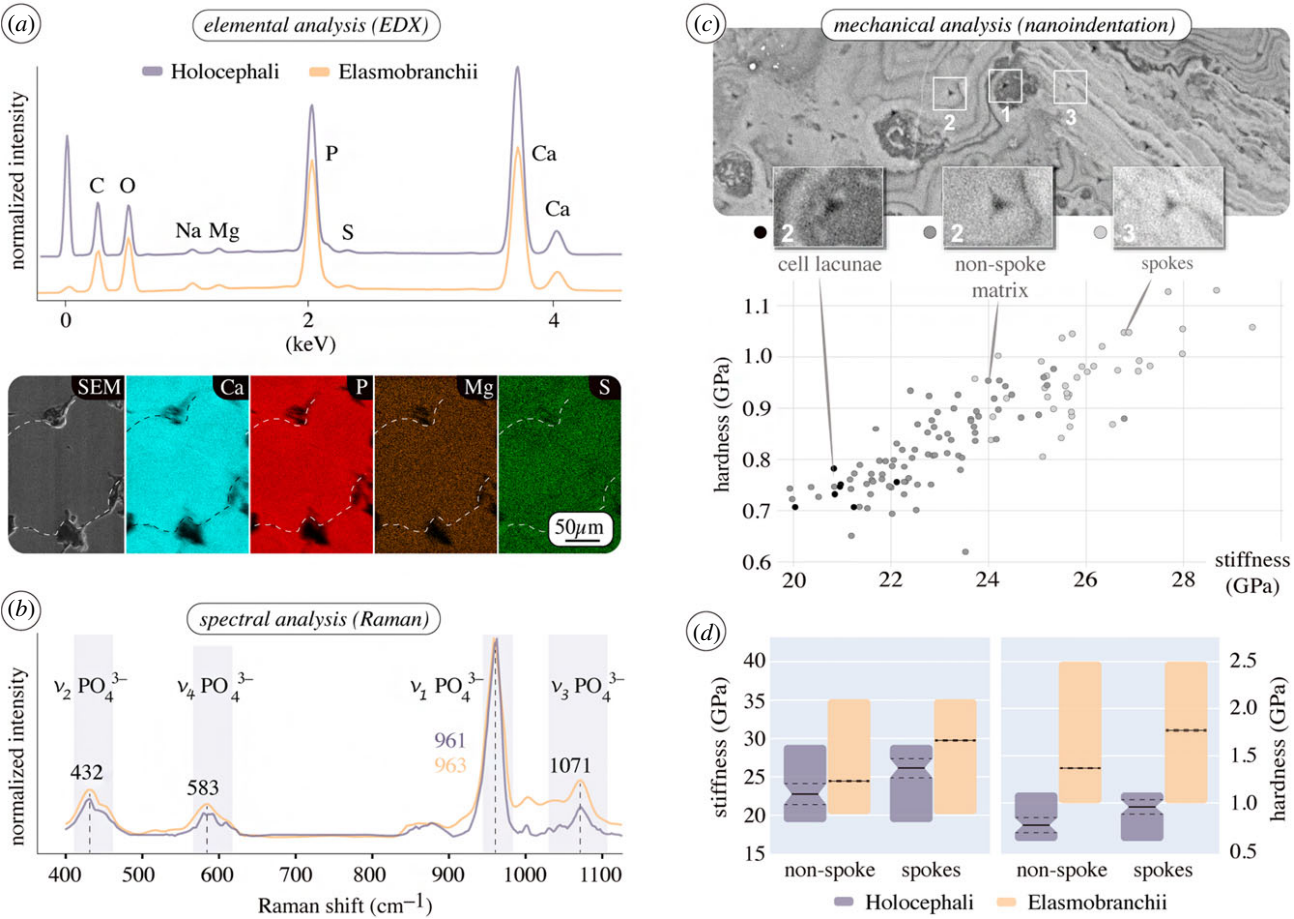
Chemical and mechanical similarity of elasmobranch and holocephalan tesserae. (*a*) Area normalized spectral graphs (EDX) of tesserae from *C. monstrosa* and round stingray *U. halleri,* and elemental maps from chimaera tesserae showing no regional variation in elemental composition. Relatively high phosphate concentrations result in low Ca/P ratios in both elasmobranch and chimaera tesserae (atomic percent: approx. 1.25 at% and approx. 1.35 at%, respectively) in comparison to bone (approx. 1.59 at%) [101]. (*b*) Raman spectra of tesserae from *U. halleri* and *C. monstrosa*, identifying the mineral in both as carbonated apatite. Raman peaks indicating vibrational bands of the phosphate group in the apatite. (*c*) Nanoindentation of *C. monstrosa* tesserae (planar section, figure 3*g*), illustrating a positive correlation of mineral density (grey value from the BSE image) and mechanical properties (indentation modulus and hardness), similar to that seen in elasmobranch tesserae [13]. (*d*) Range (coloured bars), mean (black line) and standard deviation (dashed line) of indentation modulus, stiffness (*E*) and hardness (*H*) values from non-spoke and spoke regions in planar sections of tesserae from *C. monstrosa* and *U. halleri* [13].

As in elasmobranchs, the tesserae of *C. montrosa* are composed of carbonated apatite, with large, local variation in backscatter signalling (e.g. from spokes and Liesegang lines; figure 3) due to local differences in mineral density, not elemental composition (figure 5*a*,*b*). Our micro-mechanical tests (nanoindentation on planar sections of *C. monstrosa* tesserae) showed also that mineral density correlates positively with material properties (indentation stiffness and hardness), being similar to those obtained from planar sections of elasmobranch tesserae (figure 5*c*,*d*). Like bone, elasmobranch tesserae exhibit anisotropic material properties related to preferred axes of loading and growth, being stiffer loaded along the axis of tesseral contract, than perpendicular to it [13]. Chimaera tesserae may exhibit similarly anisotropic material properties (e.g. between vertical and planar sections); this should be explored in future work, as it could provide clues to the skeletal mechanics of holocephalan fishes, for whom many aspects of ecology and behaviour remain mysterious.

## 3. Conclusion

Our findings settle a long-standing debate, demonstrating that in modern chimaera, skeletal calcification is not ‘continuous’ nor is tessellated cartilage ‘lost’, as previously suggested [84,89,90]. We show that not only is tessellated cartilage indeed a shared character between modern elasmobranchs and chimaera, but also that multiple specific morphological characters unify tessellated cartilage and tesserae in these groups. For example, patterns of mineral density variation, such as Liesegang lines and spokes, reflect common mechanisms of tesserae development and their interaction, despite great interspecies variation in tesserae shape and size. Additionally, while tesserae provide rigidity, the fibrous and flexible intertesseral joints in chimaera and elasmobranchs are likely crucial for both skeletal mechanics and development, allowing interstitial growth of the tesseral layer to keep up with the volumetric growth of the underlying unmineralized cartilage. The geometric combination of soft and hard materials—functioning under tension and compression, respectively—forms an effective and, in an evolutionary sense, also successful armour for this peculiar endoskeletal type.

The tessellation pattern in chimaera appears to be less regular than that of most shark and ray skeletons, although chimaera tesserae morphology does bear a resemblance to that of modern species of more basal modern shark lineages (e.g. horn and sevengill sharks). Tesserae in the chondrocranium of *C. monstrosa* are much smaller than those of most adult elasmobranchs we have observed; however, a careful and structured analysis of how tesseral form varies with anatomical location and phylogeny is sorely lacking. The most distinct morphological difference between chimaera tesserae and most elasmobranch tesserae is the absence of intratesseral cells in the former. Such cell populations are thought to have major implications for the active control of tissue mineralization in elasmobranchs, particularly in maintaining tesserae LCNs and the unmineralized joints between tesserae. If these hypotheses are correct, the lack of intratesseral cells in *C. monstrosa* could indicate an important physiological shift between holocephalan and elasmobranch lineages and explain the less regular tesserae shape and patterning of tessellated cartilage in chimaera. In fact, cell presence and arrangement may be the root of all the shape differences in elasmobranch tesserae, where ‘cell-rich’ tesserae tend to be large and well defined, polygonal tiles (e.g. in *Raja stellulata*, *Rhinoptera bonasus*), whereas comparatively small, irregularly shaped tesserae exhibit limited intratesseral cellular networks (e.g. in *Torpedo californica*, *H. francisci* and *N. cepedianus*) [23].

Skeletal remains from extinct chondrichthyans show that fossil species possessed mineralized, skeletal tessellations, providing strong evidence that tessellated cartilage in a general sense can be considered an ancient feature of conventionally defined chondrichthyans [104]. However, from the recent high-resolution analyses of tessellated cartilage, including the current study, it seems that the full array of ultrastructural characters seen in modern tesserae did not exist in extinct forms. This could mean that these features evolved to meet particular functional demands (e.g. associated with the feeding ecologies of modern species), but the phylogenetic placement of extinct chondrichthyans is still too muddy (and contentious) to cobble together the evolutionary sequence of feature acquisitions in tessellated cartilage. The characters we outline here that seem to unify modern chondrichthyan tesserae should be explored explicitly in extinct forms: modern features like intratesseral spokes seem to be visible, for example, in published images of tesserae from the extinct Devonian chondrichthyan, *Gogoselachus lynbeazleyae* (fig. 8C in [86]), but were not identified specifically as such. The disconnect between modern and palaeontological skeletal biology calls for stronger links among researchers in these fields, particularly for refining the skeletal character states used to define groups. By detailing salient features of modern tessellated cartilage and bringing extant chimaera into the conversation on tesserae evolution, we offer standardized terminology to support and spark the next decades of integrated research on cartilaginous fish and vertebrate skeletal evolution.

## 4. Material and methods

### 4.1. Species examined and sample preparation

All elasmobranch data reported within this article were gathered in previous studies, see [3,13,23,44]. The following workflow and protocol(s) were used to obtain data from *C. monstrosa*.

Skeletal pieces from the chondrocranium (from the roof of the mouth) were dissected from a male, adult chimaeroid fish (*C. monstrosa*) donated (from Charlie Underwood, Birkbeck College) for another study. The head of the animal was stored in a freezer at −20°C until sample preparation. The head was re-thawed in warm water, the samples were dissected and subsequently (i) fixed with PFA (see below) for histology; or (ii) dehydrated in an ascending alcohol series and stored in 75% EtOH at 4°C before measurements; or (iii) air-dried flat between two Teflon plates to prevent bending.

### 4.2. Tissue preparation for histology

After fixation with 4% PFA in phosphate-buffered saline (PBS, 0.1 M) for 24 h, the samples were stored in PBS (0.1 M, 0.05% sodium azide) and shipped to the Medical University of Innsbruck, Austria, for light microscopy (LM) and transmission electron microscopy (TEM) imaging.

### 4.3. Light microscopy

The samples were decalcified with ethylenediaminetetraacetic acid (EDTA) for one week, dehydrated with a graded isopropanol and xylene series, and embedded in paraffin using a routine histological infiltration processor (Miles Scientific Inc., Naperville, IL, USA). Serial cross-sections (7 µm) were made on a HM 355 S microtome (Microm, Walldorf, Germany), and three sections per slide mounted on SuperFrost^®^Plus slides. Sections were stained with haematoxylin and eosin (HE) (Shandon Varistain 24-4, HistocomVienna, Austria). They were examined with a Zeiss Axioplan 2 (Zeiss, Oberkochen, Germany) and photographed as colour images using a Zeiss AxioCam HR and AxioVision 4.1. software running on a Pentium 4 (Intel Inc., Santa Cruz, CA,USA) with WindowsXP (Microsoft Inc., Redmond, WA, USA).

### 4.4. Transmission electron microscopy

The samples were postfixed with 0.5% osmium tetroxide in distilled water for 12 h at 4°C, decalcified as described before and embedded in EPON resin. Semithin sections (1 µm) were cut on a Reichert Ultracut S microtome (Leica Microsystem, Wetzlar, Germany) with a histo-jumbo-diamond knife (Diatome, Biel, Switzerland) [105] and stained with toluidine blue for 1 min at 60°C. Serial ultrathin sections (90 nm) were cut with the same microtome with an ultra-diamond knife (Diatome, Biel, Switzerland), and mounted on dioxan-formvar coated slot-grids (#G2500C, Christine Gröpl, Elektronenmikroskopie, Tulln, Austria). The sections were stained with uranyl acetate and lead citrate (Leica Ultrostainer Leica Microsystem, Wetzlar, Germany). Sections were examined with a Philips CM 120 transmission electron microscope at 80 kV (FEI, Eindhoven, The Netherlands) equipped with a MORADA digital camera (Olympus SIS, Münster, Germany).

### 4.5. X-ray micro-computed tomography

Tomographic data were obtained using an EasyTom Nano 160 (RX Solutions, Chavanod, France). High-resolution µCT scans were performed with air-dried, non-embedded samples, with minimum isometric voxel sizes of 452 nm, at 60 kV source voltage and 200 µA source current. Multiple non-embedded, wet samples (75% EtOH) were scanned at isometric voxel sizes of 1–3.5 µm, at 60–80 kV source voltage and 120–200 µA source current to confirm analytical conclusions. The segmentation and imaging of the CT data were done in Amira-Avizo (Thermo Fisher Scientific) using different rendering and slicing modules. The µCT images in figure 2 are obtained from air-dried, non-embedded samples.

### 4.6. Tissue preparation for other methods

Backscatter SEM, nanoindentation and Raman spectroscopy data were obtained from air-dried samples. The air-dried samples were mounted (not embedded) on object slides using double-faced adhesive tape, then polished by hand with sandpaper plates with descending grain sizes. For final polishing, a soft polishing plate with diamond spray (0.25 µm grain size) was used.

### 4.7. Backscatter scanning electron microscopy and energy dispersive X-ray spectroscopy

BSE microscopy allows the imaging of either changes in tissue elemental density or composition as greyscale variation. Images were acquired using a field emission-environmental scanning electron microscope (FE-ESEM, FEI Quanta 600F) in environmental mode (i.e. at low vacuum without sputtering) with acceleration voltage of 10–12.5 kV. To determine the nature of the greyscale variation observed in BSE, we performed EDS using a JEOL JSM 7500F scanning electron microscope equipped with two Oxford X-Max 150 silicon drift detectors. Using EDS, we analysed the elemental composition of tesserae from *C. monstrosa* with regard to elements relevant to mineral formation (calcium, magnesium, sodium, phosphorus and sulfur). All EDS spectra and elemental maps were acquired at 15 kV acceleration voltage and 10 µA emission current, and paired with BSE images of the same regions of interest.

### 4.8. Raman spectroscopy

Raman spectra were acquired using a confocal Raman microscope (CRM200, WITec GmbH, Ulm, Germany) equipped with a P-500 piezoscanner (Physik Instrumente, Karlsruhe, Germany) and a CCD sensor (Princeton Instruments Inc., Trenton, NJ, USA). A 532 nm laser was used to generate Raman scattering while minimizing autofluorescence and the resulting spectra were investigated using WITec Project software (v. 2.10, WITec GmbH, Ulm, Germany). An integration time of 1 s and an accumulation number of 60 were used for acquisition.

### 4.9. Nanoindentation

Load-controlled nanoindentation studies were performed using a TriboIndenter (Bruker-Hysitron, MN, USA) equipped with a standard 2D transducer and a Berkovich diamond indenter. The diamond tip was calibrated using a standard fused quartz sample prior to the experiments. A load function of 5 s–2 s–5 s (loading, holding and unloading) with a maximum load of 2 mN was used for the measurements. A total number of 180 indents were performed for the measurements. The Oliver–Pharr method was used for the calculation of the elastic modulus and hardness of the samples.

## Data accessibility

All data are long-term stored at Max Planck Institute of Colloids and Interfaces, Postdam-Golm (Germany), and can be found in the following links: https://dx.doi.org/10.17617/3.4t or https://edmond.mpdl.mpg.de/imeji/collection/cOhHQ5_omblEk4xU.
